# To update or not to update the ESCMID guidelines for the diagnosis and treatment of biofilm infections – That is the question! The opinion of the ESGB board

**DOI:** 10.1016/j.bioflm.2023.100135

**Published:** 2023-07-03

**Authors:** Niels Høiby, Claus Moser, Antonio Oliver, Craig Williams, Gordon Ramage, Elisa Borghi, Joana Azeredo, Maria Dolores Macia, for the ESGB board

**Affiliations:** a European Society for Clinical Microbiology and Infectious Disease Study Group for Biofilms (ESGB), Denmark; b Department of Clinical Microbiology, Rigshospitalet, University of Copenhagen, Denmark; c Institute of Immunology and Microbiology, Panum Institute, University of Copenhagen, Denmark; d Servicio de Microbiologia, Hospital Universitario San Espases, Instituto de Investigación Sanitaria de les Illes Balears, Palma, Palma de Mallorca, Spain; e School of Medicine, Densistry & Nursing, College of Medical, Veterinary and Life Science, University of Glasgow, Glasgow, Scotland, UK; f Department of Health Sciences, Università degli Studi du Milano, Milan, Italy; g Centro de Engenharia Biológica, Universidade do Minho, Braga, Portugal

## Abstract

**Background:**

The work on the ESGB guidelines for diagnosis and treatment of biofilm infections began in 2012 and the result was published in 2014. The guidelines have been and still are frequently cited in the literature proving its usefulness for people working with biofilm infections. At the ESGB Biofilm conference in Mallorca 2022 (Eurobiofilms2022) the board of the ESGB decided to evaluate the 2014-guidelines and relevant publications since 2014 based on a lecture given at the Eurobiofilms2022.

**Guideline methods:**

The Delphi method for working on production of guidelines and the current ESCMID rules for guidelines are presented. The criteria for evaluation of relevant literature are very strict and especially for treatment, most clinicians and regulatory authorities require convincing results from Level I (randomized controlled trials) publications to justify changes of treatments. The relevant new biofilm literature and the relevant biofilm presentations from the Eurobiofilms meetings and ECCMID conferences was used for evaluating the contemporary relevance of the ESGB 2014 guidelines.

**Diagnosis of biofilm infections:**

Several infectious diseases have been recognized as biofilm infections since 2014, but the diagnostic methods and therapeutic strategies are still the same as recommended in the 2014 ESGB guidelines which are summarized in this opinion paper.

**Treatment of biofilm infections:**

Some promising new *in vitro* and *in vivo* (animal experiments) observations and reports for therapy of biofilm infections are mentioned, but they still await clinical trials.

**Conclusion:**

The interim opinion at the present time (2022) is therefore, that the guidelines do not need revision now, but there is a need for survey articles discussing new methods of diagnosis and treatment of biofilm infections in order - hopefully – to give inspiration to conduct clinical trials which may lead to progress in diagnosis and treatment of patients with biofilm infections.

## 1 Historical context of the ESGB guidelines for diagnosis and treatment of biofilm infections 2014

The ESCMID Study Group for Biofilm infections (ESGB) was proposed by Niels Høiby and colleagues at a meeting during the 15th ECCMID in Copenhagen, April 2nd-7th, 2005. One of the purposes of the ESGB was to produce guidelines for diagnosis and treatment of biofilm infections. ESGB applied for and received a one-year ESCMID grant for this purpose September 24th, 2012, and the work began November 20th-22nd, 2012 at the Panum Institute, University of Copenhagen. The Delphi method was used and preformed written questionaires were mailed to the participants and discussed and answered during the work at the Panum Institute where two subgroups worked on their part of the questions and the answers were then discussed in plenum until agreement was reached. A preliminary manuscript was written and mailed to the participants and corrected until uniform agreement was reached. All the participants became authors of the guidelines. Thereafter the final manuscript was sent to the board of ESCMID who organized peer reviews whereafter the manuscript was modified and submitted to ESCMID. The guidelines were accepted by ESCMID October 14th, 2014, and published as a CMI supplementum online January 14th, 2015 [1]. The guidelines were presented at a very well attended session during the 25th ECCMID in Copenhagen April 24th-27th, 2015, (and also at the 26th ECCMID in Amsterdam 2016) 10 years after the formation of ESGB, which during these years had started many other activities *e. g.* bi-annual European biofilm conferences beginning in 2007 in Mallorca.

The criteria used by the ESGB for evaluation of the evidence from the literature about biofilm infections were already approved by ESCMID and are shown in Table 1S (Supplementary data). These criteria have been used previously in the area of cystic fibrosis where Høiby had participated in the preparation of guidelines [2]. The guidelines in the cystic fibrosis area have not been updated by the European Cystic Fibrosis Society, but complimentary guidelines involving new areas have subsequently been produced [3].

The present ESCMID organization for preparing of guidelines was not existing in 2012–14 but the work of the ESGB was scientifically attractive for the participants. This has, however, changed in the following years as ESCMID continued to grow and to collaborate with other large international medical organizations, and subsequently became member of the Guidelines International Network that has built an international guideline library containing around 4000 guidance documents. These documents have mainly been developed or endorsed by the organizational members and are available for organization members only. To get access, one is asked to contact the ESCMID Office (guidancedocuments(at)escmid.org). ESCMID has established a guideline subcommittee with eight members headed by a guideline director and the subcommittee has (April 8th, 2022) produced version 3 of its Manual for Clinical Practice Guidelines and other Guidance Documents. This manual consists of 52 pages and includes 5 references, whereas the ‘ESCMID guidelines for the diagnosis and treatment of biofilm infections 2014’ consists of 25 pages and 214 references. In the ESCMID Library there are 35 guidelines 2017-22 but none before 2017. The ESGB guidelines for the diagnosis and treatment of biofilm infections 2014 is present in the information about ESGB in the ESCMID homepage and in PubMed and can be downloaded for free.

## 2 Is it time for update of the guidelines?

Since its publication, the biofilm guideline document has been cited 683 times (May 16th, 2023) starting in 2015 (32 citations) and reaching about 100 citations per year since 2019, which shows there is a clear interest in these guidelines in the biofilm community. However, as clinical biofilm research is a fast-moving field, it may be time to consider an update. The relevant new (since 2015) biofilm literature and the relevant biofilm presentations from the Eurobiofilms meetings and ECCMID conferences was used for evaluating the contemporary relevance of the ESGB 2014 guidelines. Guidelines are, however, published by other societies for specified biofilm infections *e.g.* definitions of periprostetic joint infections in 2021 by the European Bone and Joint Infection Society (EBJIS) supported by the ESCMID Study Group for Implant-Associated Infections [4] and such focused or supplementary non-ESGB biofilm guidelines are important, since they underline the growing understanding of the importance of biofilm infections.

### 2.1 Some recently described biofilm infections

Several infections have been recognized as surface-adhering or non-adhering biofilm infections since 2014. Non-adhering biofilm infections (aggregates in tissue or secretions) are more difficult to find and recognize as biofilm infections due to the small size of the biofilms [5] compared to adhering biofilm infections, but some examples of recently recognized non-adhering biofilm infections are *Mycobacterium abscessus* lung infections in cystic fibrosis patients [6], hidrosadenitis in humans [7], *Borrelia* lymphocytomas [8] and maybe Crohn's disease in humans which resembles Johne's disesase in cattle [9]. Other chronic infectious diseases e.g. chronic *P. aeruginosa* lung infection in patients suffering from bronchiectasis or cilia dyskinesia syndrome and the fungal disease onychomycosis have also been recognized as biofilm infections as will other chronic infections probably be in the future [[10], [11], [12]].

### 2.2 Diagnosis of biofilm infections

The principles and methods for diagnosing biofilm infections which were described in the 2014-ESGB guidelines [1] are shown in Table 2S (Supplementary data). Unfortunately, there are no new validated methods, which expand the number of methods shown in Table 2S and some clinical microbiology laboratories may not yet employ methods for improved biofilm detection such as sonication to release adhering biofilms from *e.g*. artificial joints or indwelling catheters and thorough microscopy of tissue samples. Concerning microscopy, the guidelines for diagnostic microscopy of sputum for *M. tuberculosis* is to examine at least 100 high power fields for approximately 5 min before recording a negative result because there may be very few *M. tuberculosis* cells present in sputum from some patients [13]. Such an approach may also be useful for detecting small and rare aggregates of biofilm bacteria in clinical samples [5,13].

### 2.3 Prevention and treatment of biofilm infections

**Fig. 1:**
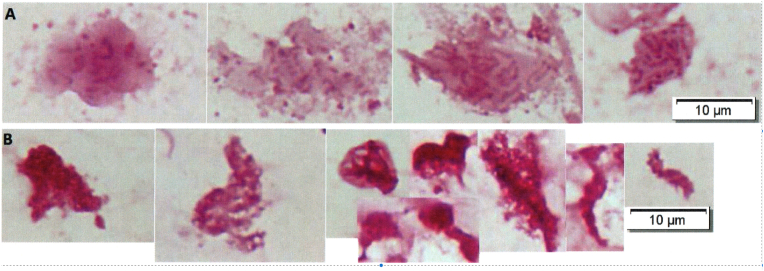
Gram stains of sputum from a cystic fibrosis patient with chronic *P. aeruginosa* biofilm lung infection before (A) and after (B) a 2-week course of suppressive antibiotic therapy intravenously and nebulised. Gram-negative rods in aggregates embedded in slime from sputum preparations made with the same sputum samples from which CFU was measured before (10^8^ CFU/ml) and after (10^3^ CFU/ml) treatment which resulted in increase of lung function (FEV1) from 58% to 85% predicted. Note that the biofilm looked more condensed but persisted (B) in spite of significant decrease of CFU and clinical improvement of the patient. This is in agreement with antibiotic killing of the surface located bacteria in the biofilms [16,32,33]. (From ref. 17, reproduced by permission of the authors and publisher).

**Fig. 2:**
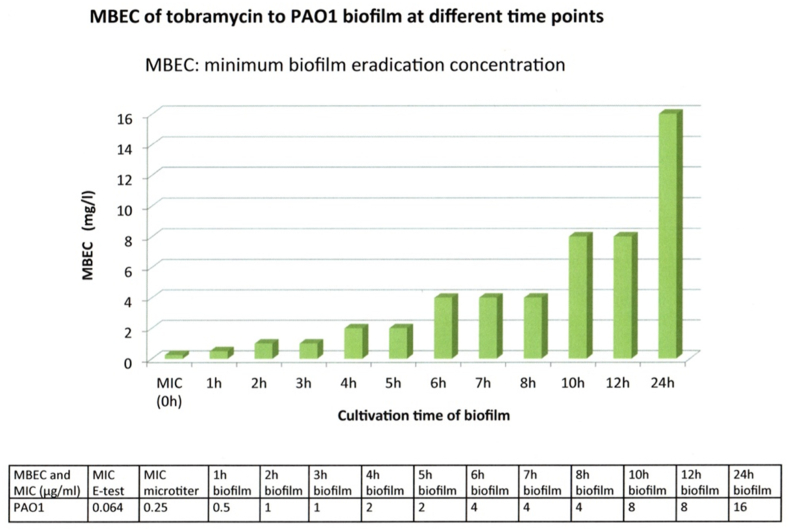
Minimum biofilm eradication concentration (MBEC) determined using the Calgary Biofilm Device [18]. The MBEC was determined at the specified time (h) after the start of biofilm growth of *P. aeruginosa* PAO1 [15]. MIC: minimum inhibitory concentration [15]. (From ref. 15, reproduced by permission of the authors and publisher).

Since biofilm growing bacteria are physiologically *tolerant* to antibiotics, the results obtained by antibiotic susceptibility testing of planktonically growing bacteria cannot be used for predicting therapeutical success of antibiotic treatment of biofilm infections with respect to eradication of the infection [1,[14], [15], [16]] although temporary clinical improvement may occur [17](Fig. 1). Use of the Calgary devise employing surface attached biofilms has, unfortunately, not solved the problem [18] and does not comprise non-attached biofilms (small aggregates in tissues or secretions) [5]. Susceptibility testing of planktonically growing bacteria (by *e.g.* the EUCAST method) uses a well-defined inoculum of planktonic cells (MacFarlane 0.5 = 10^8^ CFU/ml), however, *e.g. Mycobacterium abscessus* and other mycobacteria grow as biofilm aggregates both in fluid media and on solid media and the sizes of the aggregates increase with duration of their growth [19]. Therefore, variable results of susceptibility testing become a problem which to some degree can be reduced by dissolving the aggregates with TWEEN 80 [19] but it does not solve the problem of predicting clinical success of treatment [20,21]. This problem is illustrated by Fig. 2 which shows the increase of tolerance with duration of the growth of an adhering *P. aeruginosa* biofilm on the Calgary Biofilm Device. The consequence is that the treatment must be prolonged and continued for months or even years but still with limited success [20,21]. Presently, therefore, there is still no solution to the problem of *in vitro* susceptibility testing of biofilm growing bacteria with respect to obtain results which can predict clinical success of an antibiotic treatment of biofilm growing bacteria. An important reason for the physiological tolerance to antibiotics of biofilms is that the bacteria located in the surface of biofilms are metabolic active whereas the bacteria located in the deeper part of the biofilm are metabolic inactive and the cells are therefore dormant or grow very slowly and they therefore need much longer time of antibiotic treatment to be killed [22,23]. This is at least partly due to consumption of oxygen by the polymorphonuclear leukocytes dominated inflammation around the biofilms and by the microbial cells in the surface part of biofilms [24]. By treatment of biofilms with hyperbaric oxygen the effect of antibiotics can be improved both *in vitro* and *in vivo* in animal experiments [[25], [26], [27]] and a clinical trial of this principle is ongoing.

**Fig. 3:**
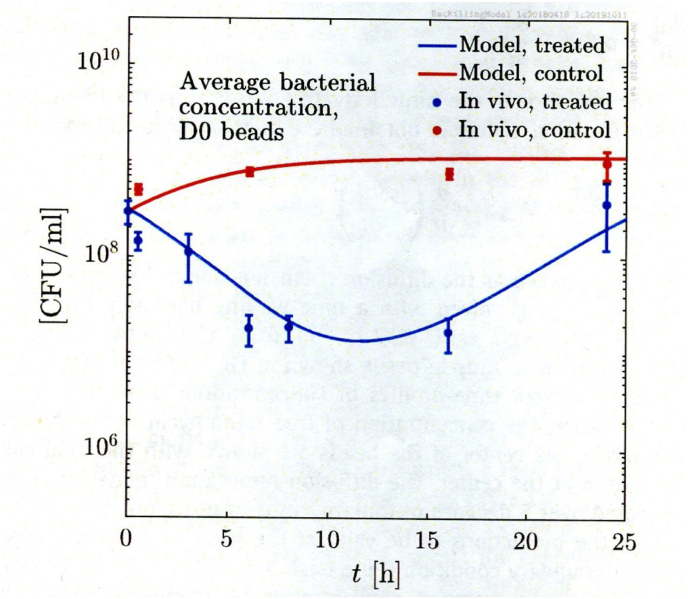
Quantitative bacteriology (CFU) inside alginate beads recovered at different time after subcutaneous installation in mice. The time points also represent the time from administration of tobramycin 40 mg/kg subcutaneously. The y-axis is log. scale. Non-treated mice (controls) are represented by red points and red line and tobramycin treated mice are represented by blue squares and blue line. Mean and SEM of CFU in all mice euthanized. The lines represent the mathematical killing model prediction [31]. (From ref. 31, reproduced by permission of the authors and publisher). (For interpretation of the references to colour in this figure legend, the reader is referred to the Web version of this article.)

The principles and methods used for antibiotic treatment of biofilm infections are shown in Table 3S (Supplementary data) [1]. The definitions used to describe the interaction of antibiotics with planktonic or biofilm growing bacteria are shown in Table 4S (Supplementary data). However, although the general pharmacokinetic/pharmacodynamic rules of antibiotic actions are the same for planktonically and biofilm growing bacteria [2], biofilms represent a 3rd compartment where the free antibiotic concentration is lower and appears delayed compared to the interstitial fluid compartment (Fig. 3) and this should be taken into account when treating biofilm infections [[28], [29], [30], [31]]. Combinations of two or more antibiotics one of which kills the metabolic active surface located bacteria in biofilms and the other kills the inactive center-located bacteria is working *in vitro* and in animal experiments [32,33], but unfortunately do not eradicate *P. aeruginosa* biofilm infections completely in cystic fibrosis patients and that may at least in part be due to the 3rd compartment problem [17,[29], [30], [31]]. New methods such as bacteriophage therapy or methods to break biofilms are also working *in vitro* and in animal experiments but await clinical trials [34,35]. Moreover, the antifungal pipeline has long been limited in options to manage fungal biofilms, but there is renewed optimism with a new panel of compounds entering the market [36].

## 3 Discussion and conclusions

The clinical influence of guidelines depends on the quality of the scientific evidence behind the recommendations. Table 1S (Supplementary data) shows, that the best evidence (Level I) is obtained from randomized controlled trials which are difficult and expensive and takes long time to perform especially concerning rare diseases. An example is the Danish multicenter POET study of partial oral definitive endocarditis antibiotic treatment [37]. Endocarditis is a biofilm infection [1] and the yearly incidence of endocarditis in Denmark is approximately 700 patients and 400 eligible patients had to be enrolled and followed for 210 days (180 days after completion of therapy). The completion of the study *per se* took six years, beginning in 2011 and was completed in 2017 and subsequently awaiting the 6 months observation period after completing of therapy (not including the substantial time to design and planning of the protocol and obtaining permissions from authorities, as well as the subsequent data handling) and was published in 2019. A 5-years long-term follow up study from time of the last randomization in 2017 was published in 2022 [38]. Therefore, the total time for this study was approximately nine years from idea and planning to the publication of the main article. Even Level II evidence takes long time to perform in many cases [39]. In the case of the POET study the change from intravenous therapy during hospitalization to partly oral therapy outside hospitals – after initial intravenous therapy in the hospitals – may seem a small scientific step for basic scientists. However, the consequence of choosing an inferior therapy may be lethal to some patients, and therefore clinicians (and the regulatory authorities) will not change their therapy unless convincing results from well-designed trials (Level I and sometimes Level II) support such changes. The ESGB is therefore encouraging such clinical trials in the fields of diagnosis, prevention and treatment of biofilm infections [1]. The Guideline Committee of the ESCMID study group of *Clostridioides difficile* published their first treatment guidance in 2009 which was updated in 2014 and again in 2021 [40]. They evaluated the effect of three different antibiotics, a toxin-binding monoclonal antibody and fecal microbiota transplantation. The updated guidance was published in 2021 and comprise 21 pages and 289 references and involved 20 authors and a librarian from 10 countries and 8 external experts from 6 countries, so even updates of guidelines for treatment of a single disease is a comprehensive task [41].

Concerning *diagnosis of biofilm infections*, the ESGB-2014 guidelines [1] are up to date, but the application of the recommended laboratory methods may not yet have been adopted in all clinical microbiology laboratories. In addition, the increasing relying on automated molecular biological methods including PCR *e.g*. multiplex PCR does not facilitate biofilm detection. Biofilm recommendation for diagnosis is still microscopy (*aggregates*), immune response (*chronic infections*), sonication to release adhering biofilms and aggregates and prolonged incubation of cultures. Unfortunately, neither culture nor molecular methods – except microscopy notably using the PNA-FISH technique [13] – can distinguish between planktonic and biofilm growing microbes. In the 2014 guidelines question 1–7 [1] deals with which research is urgently needed to improve diagnosis of biofilm infections. A long list of proposed research is given in these guidelines [1], and there seems not to be a significant need for revision.

Concerning *prevention and treatment of biofilm infections,* the ESGB-2014 guidelines are clinically up to date and include *antibiotic prophylaxis* for *e.g.* hip replacement surgery; *preemptive antibiotic treatment* of intermittent colonization if there is a risk of development of biofilm infection *e.g. P. aeruginosa* in cystic fibrosis; *removal of foreign bodies* with adhering biofilms *e.g.* intravenous access catheters or use of *antimicrobial locks* [41]; *long-term chronic suppressive antibiotic therapy* to reduce tissue damage *e.g*. for cystic fibrosis with chronic *P. aeruginosa* lung infection. In the 2014 guidelines question 2–7 [1] deals with which research is urgently needed to improve prevention and treatment of biofilm infections. A long list of proposed research is given in the guidelines [1], there seems not to be a significant need for revision.

The guidelines [1] are still adequate for clinicians and for clinical microbiologists. The new results discussed here are not ready for clinical use but deserve a comprehensive review in *e.g.* CMI or BIOFILM, highlighting promising new results which may lead to randomized controlled clinical trials or ‘off label use’ or ‘compassionate use’ in critical clinical situations.

## Conflict of interest

The authors have no conflicts of interests. There was no funding of the project.

## CRediT authorship contribution statement

**Niels Høiby:** wrote the manuscript based on his invited oral presentation at the EUROBIOFILM 2022, Mallorca, August 31st-September 3rd, 2022. **Claus Moser:** Conceptualization, Writing – review & editing. **Antonio Oliver:** Conceptualization, Writing – review & editing. **Craig Williams:** Conceptualization, Writing – review & editing. **Gordon Ramage:** Conceptualization, Writing – review & editing. **Elisa Borghi:** Conceptualization, Writing – review & editing. **Joana Azeredo:** Conceptualization, Writing – review & editing. **Maria Dolores Macia:** Conceptualization, Writing – review & editing.

## Declaration of competing interest

The authors have no conflicts of interests.

## Data Availability

No data was used for the research described in the article.
