# Supplementary material for: To update or not to update the ESCMID guidelines for the diagnosis and treatment of biofilm infections – That is the question! The opinion of the ESGB board
Source: Biofilm. 2023 Jul 3;6:100135. doi: 10.1016/j.bioflm.2023.100135 (PMC10704329; doi:10.1016/j.bioflm.2023.100135)
Supplement: Multimedia component 1 [file mmc1.docx]

**Supplementary material**

**Tables**

Table 1S. Criteria for evaluation of the evidence behind the recommendations in the ESGB guidelines for diagnosis and treatment of biofilm infections (1).

**_______________________________________________________________________________**

**Strength of a recommendation**:

Grade A: ESCMID strongly supports a recommendation for use.

Grade B: ESCMID moderately supports a recommendation for use.

Grade C: ESCMID marginally supports a recommendation for use.

Grade D: ESCMID  supports a recommendation against use.

**Quality of evidence:**

Level I: Evidence from at least one properly designed randomized controlled trial.

Level II: Evidence from at least one well-designed clinical trial, without randomization; from cohort or case-controlled analytic studies (preferable from >1 center); from multiple time series; or from dramatic results of uncontrolled experiments.

Level III: Evidence from opinions of respected authorities, based on clinical experience, descriptive case studies.

________________________________________________________________________________

**Added index**:

-Meta-analysis or systematic review of randomized controlled trials.

-Transferred evidence, that is, results from different patients' cohorts, or similar immune-status situation.

-Comparator group is a historical control.

-Uncontrolled trial.

-Published abstract (presented at an international symposium or meeting).

________________________________________ __________________________________

Table 2S. General features of clinical and laboratory indications for diagnosis of biofilm infections (1).

*Clinical features:*

- Clinical signs of infection *e.g.* the classical but frequently low-grade inflammatory reactions tumor, rubor, dolor, loss of function and sometimes low-grade fever.
- Medical history of biofilm-predisposing condition (*e.g*. implanted medical device, cystic fibrosis).
- Persisting infection lasting >7 days (this is unspecific, and other reasons are frequent such as resistance to the antibiotics used).
- Failure of antibiotic treatment and recurrence of the infection (particularly if evidence is provided that the same organism is responsible on multiple time points) - typing of the pathogen.
- Documented evidence/history of antibiotic failure.
- Evidence of systemic signs and symptoms of infection that resolve with antibiotic therapy, only to recur after therapy has ceased.

*Microbiological diagnostics:*

- Microscopic evidence from fluid/tissue samples obtained from the focus of the suspected infection:

-Microscopy revealing the presence of microbial aggregates and biofilm structure (smear or fluid sample, but ideally from tissue sample if possible).

-Microscopy revealing evidence of microbial aggregates co-localized with inflammatory cells.

-Microbiological evidence of aggregated microorganisms consistent with infectious etiology.

- Positive culture/non-culture-based techniques (PCR) of fluid or tissue sample:

-Culture-based identification of microbial pathogens (MALDI-TOF).

-Presence of mucoid colonies or small colony variants of *P. aeruginosa* in culture positive samples—which may indicate antibiotic recalcitrance.

-PCR, quantitative PCR or multiplex PCR positive results for pathogen associated with infection (*e.g*. *S. aureus* with implant, *P. aeruginosa* with cystic fibrosis).

-Fluorescence in situ hybridization positive results for known pathogen showing aggregated microoganisms.

-Non-culture-based identification of microbial pathogen (pyrosequencing, next-generation sequencing).

- Specific immune response to identified microorganism - (*e.g*. to *P. aeruginosa* antigens in

cystic fibrosis patients) if the biofilm infection has been present for more than 2 weeks.

____________________________________________________________________

Table 3S. Use of antibiotics to prevent and treat biofilm infections (1)

________________________________________________________________________________

- Prophylaxis – to prevent infection in non-infected patients (*e.g*. during hip-replacement surgery)*.
- Preemptive – eradication of colonizing bacteria where there is a risk of development of infection*.
- Empiric – clinical diagnosis of infection but etiology is unknown*.
- Definitive – based on isolated microorganism and known antibiotic susceptibility*.
- Chronic suppressive therapy – the biofilm cannot be eradicated but it can be suppressed, and the tissue damage diminished, and organ function maintained.

*the same principles are used to prevent and treat infections due to planktonically growing bacteria.

Table 4S. Definitions used to describe the interaction of antibiotics with planktonic or biofilm growing bacteria (1, 16).

________________________________________________________________________________

*Planktonically growing bacteria*

MIC*: Minimal Inhibitory Concentration = prevention of planktonic growth.

MBC**: Minimal Bactericidal Concentration = killing of 99,9% of the biofilm bacteria, the remaining bacteria are eliminated by the defense mechanisms of the immune-competent host.

*Biofilm growing bacteria*

MBIC: Minimal Biofilm Inhibitory Concentration = prevention of biofilm growth.

MBB: Minimal Biofilm Bactericidal Concentration = killing of 99,9% of the bacteria, the remaining bacteria are not eliminated by the defense mechanisms of the immune-competent host and results in recurrence of the infection – unless chronic suppressive therapy is employed.

MBEC: Minimal Biofilm Eradication Concentration = killing of all the bacteria in the biofilm.

________________________________________________________________________________

*susceptibility testing in the clinical microbiology laboratory which is reported to the clinicians.

**seldom determined in the clinical microbiology laboratory.

MBIC, MBB and MBEC are only determined for research purposes and not reported to the clinicians.
